# Functional disability associated with disease and quality-of-life parameters in Chinese patients with rheumatoid arthritis

**DOI:** 10.1186/s12955-017-0659-z

**Published:** 2017-05-02

**Authors:** Juan Ji, Lijuan Zhang, Qiuxiang Zhang, Rulan Yin, Ting Fu, Liren Li, Zhifeng Gu

**Affiliations:** 1 Department of Rheumatology, Affiliated Hospital of Nantong University, 20th Xisi Road, 226001 Nantong, People’s Republic of China; 2 School of Nursing, Nantong University, 19th Qixiu Road, 226001 Nantong, People’s Republic of China

**Keywords:** Rheumatoid arthritis, Functional disability, Quality of life, Joint mobility

## Abstract

**Background:**

As an important outcome measure among rheumatoid arthritis (RA) patients, functional disability may contribute to unemployment, loss of work productivity, and impaired quality of life. However, little is known about the risk factors of functional disability in Chinese RA patients. This study aimed (1) to examine the prevalence of functional disability in Chinese RA patients; (2) to explore factors associated with the health assessment questionnaire-disability index (HAQ-DI).

**Methods:**

A total of 101 RA patients in this cross-sectional study underwent standardized laboratory examinations and responded to the questionnaire for demographic data, the HAQ-DI for functional disability, the Compliance Questionnaire on Rheumatology (CQR) for medication adherence, the Hospital Anxiety and Depression Scale (HADS) for psychological status, and the Short Form 36 health survey (SF-36) for quality of life. Pain, grip/pinch strength, disease activity, and large joint mobility were recorded. Independent samples t-tests, chi-square analyses, and logistic regression modeling were used to analyze the data.

**Results:**

The mean ± SD age of RA patients was 54.9 ± 11.9 years. Approximately 15.8% RA patients in mainland China experience functional disability (defined as a HAQ-DI score ≥ 1). Long disease duration, pain, high disease activity, a larger number of tender and swollen joints, high C-reactive protein (CRP) level, decreased grip strength, and limitation of shoulder, elbow, wrist, knee, and ankle motion were associated with the HAQ-DI. Participants with functional disability tended to have more severe depressive symptoms and a lower quality of life compared with individuals without functional disability. Stepwise logistic regression analyses found that limitation of wrist extension (*P* = 0.001) and lower body pain (BP) score (*P* = 0.001) explained higher HAQ-DI score.

**Conclusions:**

The present study reported that functional disability was common in Chinese RA patients. A low quality of life and limitation of joint mobility had great impacts on functional disability in Chinese RA patients. Targeted and culturally sensitive interventions should be strengthened to delay the onset of disabilities of this population.

## Background

As an important outcome measure among rheumatoid arthritis (RA) patients, functional disability may contribute to unemployment, loss of work productivity, increased healthcare costs, and impaired quality of life for millions worldwide [1–4]. It has been reported that that people with RA have an increased risk of experiencing functional disability with the prolongation of the course of disease [5]. A published analysis of data estimated that the prevalence of functional disability was 71% at baseline and the incidence of disability improved significantly over time in the United States [6]. A study from China revealed that there was higher prevalence of functional disability in joints disease compared with other diseases or traffic accidents [7], and the prevalence of functional disability in RA patients was up to 50%, if they have delayed treatments for 2 years. All of these highlight the fact that functional disability must be understood in order to develop strategies to delay the onset of disabilities in RA patients. Developed in 1978, the health assessment questionnaire-disability index (HAQ-DI) remained the gold standard for measuring functional disability in RA [8, 9]. Current epidemiological evidence suggested gender [10], age [11], socioeconomic status (SES) [12], disease duration [11, 12], comorbidities [13, 14], pain [15, 16], disease activity [9, 13], laboratory indexes [17, 18], medication adherence [19], grip strength [17, 20], joints damage [9], anxiety/depression [21, 22], and quality of life [1, 4] were significantly associated with the HAQ-DI in RA patients. Only a study from Finland [20] in RA identified that joint mobility have greatest influence on the HAQ-DI. However, the association between these variables and the HAQ-DI among RA patients in mainland China have not yet been fully explored. Therefore we were specifically interested in the associations among SES, disease activity, joint mobility, psychological status, quality of life and the HAQ-DI in RA patients. The aims of this study were to explore the possible risk factors of the HAQ-DI using RA patient population from China, in order to identify warning signs for delaying the onset of disabilities of this patients. It was hypothesized that some disease and quality-of-life parameters would affect functional disability.

## Methods

### Study participants

Patients who fulfilled the 2010/2012 American College of Rheumatology (ACR) criteria for RA were recruited from the Affiliated Hospital of Nantong University from January 2016 to July 2016. 114 RA patients were consecutively invited to participate in a single-centered cross-sectional study and 101 (89%) patients took apart and completed the questionnaires in the end. Patients were excluded based on either of the following: (1) they were less than 18 years old; (2) they did not complete the questionnaires; (3) they had comorbidities (e.g., serious infections, or cardiac, respiratory, gastrointestinal, endocrine disease) that could influence disease activity; (4) they did not complete the measurements of grip/pinch strength, joint mobility, disease activity or pain. This cross-sectional study was approved by the Ethics Committee of the Affiliated Hospital of Nantong University, and a written informed consent was obtained from each RA patient.

### Primary outcome

Functional disability was evaluated by the HAQ-DI, which included 20 questions in eight subdimensions: dressing and grooming, arising, eating, walking, hygiene, reach, grip, and common daily activities. The response alternatives were 0, able without any difficulty; 1, able with some difficulty; 2, able with much difficulty; and 3, unable. The HAQ-DI score ranged from 0 to 3, with higher scores indicating more disability. Functional disability was defined as the HAQ-DI score ≥ 1 according to previous studies [6, 23].

### Independent variables

At baseline, sociodemographic and disease characteristics [including gender, age (years), body mass index (BMI) (kg/m^2^), education, income/person/month (Yuan), working status, health insurance, comorbidities, and disease duration (years)] were recorded.

Disease activity was estimated with the valid and reliable 28-joint Disease Activity Score (DAS28), incorporating 28 swollen and tender joint counts (SJC and TJC), patients’ assessment of disease activity (0–100 mm VAS, where 0 = not active at all and 100 = extremely active). We used erythrocyte sedimentation rate (ESR) to calculate DAS28 [24, 25]. Furthermore, the 100-mm horizontal VAS (0 = no pain, and 100 = most severe pain) was used to assess the patients’ pain [15].

Several serological markers, ESR, C-reactive protein (CRP), and rheumatoid factor (RF) were examined at the time of diagnosis. Plasma and serum samples were stored at −70 °C until assayed. ESR and CRP were assessed using the westergren method (mm/h) and the nephelometric method (mg/l), respectively. RF was measured by immunoturbidimetry using Cobas integra RFII (Roche Diagnostics GmbH, Mannheim, Germany) [26]. The common cut-off value (20 IU/ml) was adopted based on previous study [27].

Medication adherence was assessed using the Compliance Questionnaire on Rheumatology (CQR). The CQR is a 19-item, self-administered questionnaire, and was developed to correctly identify patients who were classified as “low” adherers (taking < 80% of their medication correctly). The questions were identified through focus groups and clinician’s expert opinion of the likely barriers to medication taking. The four point Likert answering scale ranged from; “Definitely don’t agree” (scored 1) to “Definitely agree” (scored 4); items 4, 8, 9, 11, 12, and 19 have to be reversely recoded (4 = 1, 3 = 2, etc.). Lower scores indicated lower levels of adherence. The CQR was validated against Medication Event Monitoring System and found to correctly identify 62% of low adherers without the extensive time and costs which are combined with “gold standard” medication monitoring techniques, such as pill counting or blood chemistry levels [28–30].

The Hospital Anxiety and Depression Scale (HADS), a 14-item questionnaire, was used to assess levels of anxiety (7 items) and depression (7 items). Each item had a 4-point Likert scale and was scored between 0 and 3, e.g., “I can sit at ease and feel relaxed,” with responses of 0 = definitely, 1 = usually, 2 = not often, and 3 = not at all. Anxiety and depression were scored separately using the 7-item subscales. Scores for each subscale were constructed by summation, ranging 0–21. Each subscale was categorized as follows: normal (0–7), and abnormal (8–21) [31]. The Chinese version of HADS had acceptable internal consistency and test-retest reliability, with a Cronbach alpha of 0.85 and intraclass correlation coefficient of 0.90, respectively [32].

Participants’ health status was assessed using the Short Form 36 health survey (SF-36). It assessed eight domains (scores ranged from 0 to 100, with higher scores indicating better health status): physical functioning (PF), role physical (RP), bodily pain (BP), general health (GH), vitality (VT), social functioning (SF), role emotional (RE), and mental health (MH). Z-transformed and normalized domain scores were grouped into Physical Component Summary (PCS) and Mental Component Summary (MCS). The questionnaire was culturally adapted and translated into Chinese. “Convergent validity and discriminant validity were satisfactory for all except the social functioning scale. Cronbach’s á coefficients ranged from 0.72 to 0.88 except 0.39 for the social functioning scale and 0.66 for the vitality scale. Test-retest reliability coefficients ranged from 0.66 to 0.94” [33].

Hand grip and pinch strength were measured with a hydraulic hand grip and pinch dynamiter (Jamar model J00105, Lafayette Instrument). The mean force (N) produced by the right and left hands of each patient was used for analysis [34].

Two experienced physical therapists measured range of motion (ROM) of the following joint actions, using a manual goniometer to within a level of accuracy of 5°in standardized positions: wrist flexion and extension; shoulder flexion and shoulder abduction; elbow, hip, and knee flexion; ankle dorsiflexion and plantarflexion [20]. The mean degree produced by the right and left sides of each patient was used for analysis.

Questionnaires and other assessments were administered to participants from January 2016 to July 2016. Written questionnaires were provided on paper, and all participants completed the questionnaires under a physician’s supervision in a clinical setting. Pain, grip/pinch strength, joint mobility, and disease activity were evaluated by the same clinician for all patients. Nurses counted results. The results were added to a computer database by two research assistants and double checked against the original data prior to analysis.

### Statistical analysis

Descriptive analyses were performed to investigate the participants’ characteristics. The data were expressed as the mean ± SD for continuous variables and as frequencies (%) for categorical variables. Variables used in this study were screened of the group difference (functional disability versus non-functional disability according to the HAQ-DI score) using univariate tests (chi-square tests and independent sample t-tests), at a lenient level of significance without correction for multiple testing (alpha = 0.05). Stepwise logistic regression analyses were conducted for functional disability to explore the significant predictors of dimorphic concerns. All variables with a significant association with functional disability by univariate tests were entered into a stepwise logistic regression model with the dichotomous functional disability measured by the HAQ-DI score as the dependent variable. Statistical significance was considered when *P* < 0.05 (two-sided). All analyses were performed using SPSS version 20.0.

## Results

As 13 RA patients did not complete the questionnaires, 101 RA patients (16 males and 85 females) were enrolled in the current study. Table 1 presented the baseline participant characteristics included in our analysis. The mean ± SD age of the respondents was 54.9 ± 11.9 years, and 84.2% were female, respectively. Almost 15.8% (*n* = 16) individuals in the sample group reported functional disability. The patients were divided into two groups according to the HAQ-DI score. For the HAQ-DI score ≥ 1 patients, significant associations were found with long disease duration (*P* = 0.042), sever pain (*P* < 0.001), a large number of tender joints (*P* = 0.013) and swollen joints (*P* = 0.001), high disease activity (*P* < 0.001), high CRP level (*P* < 0.001), medication adherence (*P* = 0.024), and grip strength (*P* = 0.016). With regard to joint mobility, wrist extension (*P* < 0.001), shoulder flexion (*P* = 0.001), elbow flexion (*P* = 0.006), knee flexion (*P* = 0.014), and ankle plantarflexion (*P* = 0.017) were significantly associated with the HAQ-DI. However, no statistically significant associations were found with regard to gender (*P* = 0.715), age (*P* = 0.904), BMI (*P* = 0.089), education (*P* = 0.755), income/person/month (*P* = 0.454), working status (*P* = 0.512), health insurance (*P* = 0.938), comorbidities (*P* = 0.425), ESR (*P* = 0.077), RF positivity (*P* = 0.346), pinch strength (*P* = 0.205), shoulder abduction (*P* = 0.063), hip flexion (*P* = 0.304), and ankle dorsiflexion (*P* = 0.910).

**Table 1:** Differences between demographic and clinical factors of non-functional disability and functional disability patients

Variables	Overall sample	Non-functional disability (HAQ-DI score < 1)	Functional disability (HAQ-DI score ≥ 1)	*P*-value
Female gender, no. (%)	85 (84.2)	72 (84.7)	13 (81.2)	0.715
Age, years	54.9 ± 11.9	54.9 ± 12.2	55.3 ± 10.3	0.904
BMI, kg/m^2^	22.5 ± 3.1	22.3 ± 2.9	23.7 ± 3.6	0.089
Education, years, no. (%)				0.755
< 9 years	76 (75.2)	63 (74.1)	13 (81.2)	
≥ 9 years	25 (24.8)	22 (25.9)	3 (18.8)	
Income/person/month, no. (%)				0.454
≤ 1000 Yuan	54 (53.5)	43 (50.6)	11 (68.8)	
1000–3000 Yuan	36 (35.6)	33 (38.8)	3 (18.8)	
3000–5000 Yuan	7 (6.9)	6 (7.1)	1 (6.2)	
≥ 5000 Yuan	4 (4.0)	3 (3.5)	1 (6.2)	
Working status, no. (%)				0.512
Employed	21 (20.8)	19 (22.4)	2 (12.5)	
Unemployed	80 (79.2)	66 (77.6)	14 (87.5)	
Health insurance, yes, no. (%)	65 (64.4)	55 (64.7)	10 (62.5)	0.938
Comorbidities, yes, no. (%)	53 (52.5)	43 (50.6)	10 (62.5)	0.425
Disease duration, years	8.0 ± 8.4	7.1 ± 7.7	13.1 ± 10.5	0.042*
VAS pain score (range 0–100)	42.6 ± 26.2	37.6 ± 23.9	69.1 ± 22.4	<0.001**
TJC (28 joints)	4.2 ± 6.1	3.1 ± 4.9	9.5 ± 9.0	0.013*
SJC (28 joints)	2.5 ± 3.5	2.0 ± 3.2	5.1 ± 4.1	0.001**
DAS28	3.5 ± 1.5	3.2 ± 1.4	4.9 ± 1.6	<0.001**
ESR, mm/h	25.7 ± 25.6	23.8 ± 23.7	36.1 ± 33.1	0.077
CRP, mg/l	15.6 ± 24.4	14.1 ± 23.4	23.6 ± 28.6	<0.001**
RF positivity, yes, no. (%)	77 (76.2)	63 (74.1)	14 (87.5)	0.346
Medication adherence, yes, no. (%)	40 (39.6)	37 (43.5)	3 (18.8)	0.024*
Grip strength ^ǂ^ (kg)	13.9 ± 9.0	14.9 ± 9.1	9.0 ± 6.7	0.016*
Pinch strength ^ǂ^ (kg)	3.3 ± 2.3	3.4 ± 2.3	2.7 ± 1.9	0.205
Wrist flexion ^ǂ^ (degrees)	39.7 ± 16.6	42.8 ± 14.7	23.3 ± 17.0	<0.001**
Wrist extension ^ǂ^ (degrees)	36.3 ± 15.4	39.8 ± 13.0	18.3 ± 14.9	<0.001**
Shoulder flexion ^ǂ^ (degrees)	129.0 ± 29.4	133.1 ± 26.0	107.1 ± 37.1	0.001**
Shoulder abduction ^ǂ^ (degrees)	126.6 ± 30.2	130.1 ± 25.9	107.8 ± 43.4	0.063
Elbow flexion ^ǂ^ (degrees)	124.2 ± 26.5	127.3 ± 23.2	107.8 ± 36.4	0.006**
Hip flexion ^ǂ^ (degrees)	77.0 ± 18.5	78.1 ± 17.0	71.2 ± 24.9	0.304
Knee flexion ^ǂ^ (degrees)	98.5 ± 27.3	102.9 ± 21.9	75.0 ± 39.7	0.014*
Ankle dorsiflexion ^ǂ^ (degrees)	12.3 ± 10.2	12.3 ± 10.6	12.0 ± 8.3	0.910
Ankle plantarflexion ^ǂ^ (degrees)	44.3 ± 14.7	45.8 ± 14.2	36.4 ± 14.9	0.017*

Values are presented as the number (%) or the mean ± SD. *HAQ-DI* the health assessment questionnaire-disability index, *BMI* body mass index, *VAS* visual analog scale, *TJC* tender joint count, *SJC* swollen joint count, *DAS28* Disease Activity Score in 28 joints, *ESR* erythrocyte sedimentation rate, *CRP* C-reactive protein, *RF* rheumatoid factor. ^ǂ^ Mean of right and left sides. *P < 0.05, **P < 0.01

An analysis found that the prevalence of depression in individuals with the HAQ-DI score ≥ 1 were significantly higher compared with individuals with the HAQ-DI score < 1. Furthermore, statistically significant associations were found with regard to all dimension scores of SF-36 (Table 2).

**Table 2:** Differences between psychological status and quality of life of non-functional disability and functional disability patients

Variables	Overall sample	Non-functional disability (HAQ-DI score < 1)	Functional disability (HAQ-DI score ≥ 1)	*P*-value
HADS-anxiety, yes, no. (%)	47 (46.5)	36 (42.4)	11 (68.8)	0.061
HADS-depression, yes, no. (%)	35 (34.7)	24 (28.2)	11 (68.8)	0.003**
Domains of SF-36				
PCS	44.5 ± 23.1	48.8 ± 21.9	21.0 ± 14.0	<0.001**
MCS	53.3 ± 22.5	56.7 ± 21.7	33.8 ± 16.6	<0.001**
PF	59.4 ± 29.4	65.2 ± 25.5	27.2 ± 29.8	<0.001**
RP	25.2 ± 39.8	29.1 ± 42.1	3.1 ± 8.5	<0.001**
BP	51.1 ± 26.2	56.7 ± 23.8	21.4 ± 18.6	<0.001**
GH	42.4 ± 20.6	44.2 ± 20.0	32.1 ± 22.2	0.031*
VT	51.7 ± 18.4	53.6 ± 17.5	39.7 ± 19.8	0.005**
SF	62.6 ± 27.4	68.1 ± 25.0	32.8 ± 20.9	<0.001**
RE	37.5 ± 45.0	41.6 ± 45.4	14.6 ± 34.3	0.011*
MH	61.4 ± 19.0	63.7 ± 17.8	48.0 ± 21.2	0.002**

Values are presented as the number (%) or the mean ± SD. *HAQ-DI* the health assessment questionnaire-disability index, *HADS* Hospital Anxiety and Depression Scale, *SF-36* Short Form 36 health survey, *PCS* physical components summary, *MCS* mental components summary, *PF* physical functioning, *RP* role limitations due to physical problems, *BP* body pain, *GH* general health perception, *VT* energy/vitality, *SF* social functioning, *RE* role limitations due to emotional problems, *MH* mental health. *P < 0.05, **P < 0.01

Stepwise logistic regression analyses were used to identify a model to predict RA patients who would have functional disability. The results indicated that limitation of wrist extension and lower BP score were significant risk factors for functional disability (Table 3). This model had a good fit under the Hosmer-Lemeshow goodness-of-fit test (χ^2^ = 1.355, *P* = 0.995). A one unit decrease in BP score was associated with the increase of functional disability (OR = 0.929, *P* = 0.001); a one unit decrease in wrist extension was associated with the increase of functional disability (OR = 0.903, *P* = 0.001).

**Table 3:** Result of analysis of stepwise logistic regression models in functional disability patients

Variables	B	S.E	*P*-value	OR (95% CI)
BP	−0.074	0.022	0.001**	0.929 (0.890, 0.969)
Wrist extension ^ǂ^ (degrees)	−0.102	0.031	0.001**	0.903 (0.849, 0.960)

*OR* odds ratio, *CI* confidence interval, *BP* body pain. ^ǂ^ Mean of right and left sides. **P < 0.01

## Discussion

This study provided evidence that functional disability was common in Chinese RA patients. We found that the risk of functional disability increases with high disease activity and limitation of joint mobility. Importantly, we also found that functional disability were significantly associated with patients’ depressive symptoms and quality of life.

The current study reported that almost 15.8% of RA patients had functional disability (HAQ-DI score ≥ 1), which was much lower than other study [6]. It could be explained by some cultural features which may influence disease management in China. Previous studies have reported that gender [10], age [11, 16], SES [12], comorbidities [13, 14], and pinch strength [17, 20] were associated with the HAQ-DI in RA patients. However, our study demonstrated there were no association between these variables and the HAQ-DI. One possible explanation for the different results was the existence of cultural diversity and the different participants included in different studies with either Chinese or Western cohorts. Not surprisingly, disease duration was significantly associated with functional disability in Chinese patients with RA, which was in line with previous studies [11, 12]. When RA progresses, the joints were increasingly affected, which possibly increased the HAQ-DI score. To our knowledge, TJC, SJC and CRP were important subdimensions of disease activity in RA, and pain is the leading reason for patients seeking medical care [35, 36]. Our study demonstrated significant associations among TJC, SJC, CRP, DAS28, pain and the HAQ-DI, which was in accordance with the previous studies [9, 10, 15–18, 20]. These could be explained that pain and disease activity were independent risk factors for the functional disability in RA patients. Furthermore, we found that there were significant association between medication adherence and functional disability, which might be explained that RA patients with reduced medication adherence could have high disease activity [37, 38]. Damage in cartilage and bony structures, narrowing of joint space, increased intra-articular liquid volume, swelling of soft tissues around the joints, and possible subsequent subluxation are important factors contributing to decreased grip strength and limited joint mobility in RA. There is a paucity of literature focusing on the association between joint mobility and the HAQ-DI in people with RA [39, 40]. In accordance with Hakkinen A and his colleagues’ finding [20], we reported that grip strength and alterations in the individual joint mobility have great influences on the HAQ-DI. This result suggested that medical care personnel should pay attention to grip strength and the individual joint mobility measurements, to develop strategies to improve their physical function. Furthermore, this study revealed RA patients with poor quality of life tended to suffer from functional disability. Our findings corroborated previous studies [1, 4], which showed that poor quality of life was significantly associated with functional disability in RA patients.

To identify which variables were most significantly correlated with functional disability, a stepwise logistic regression analysis was used. Only independent variables individually associated with functional disability with a *P*-value < 0.05 were entered into a stepwise logistic regression model. We found that low BP score and limited wrist extension were significantly associated with functional disability, which indicated that a low quality of life and decreased joint mobility were independent risk factors for functional disability.

However, this study has some limitations. First, the sample size was relatively small and the single-center study design might mean that results were not necessarily generalizable to a broader population. Second, no causal conclusions could be inferred because the study was cross-sectional in design. Another limitation of this study was the lack of data on radiographic damage in peripheral joints, since these were not assessed in this study. But previous study had found that scores for the functional disability in patients with RA were correlated at higher levels with joint mobility than with radiographic score [20]. It was not clear whether the functional disability described in this study was mainly related to joint destruction subpopulation of joint mobility or not. This question would have to be addressed in additional studies.

## Conclusions

This study reported that functional disability was common in Chinese RA patients. A low quality of life and limitation of joint mobility had great impacts on functional disability in Chinese RA patients. Targeted and culturally sensitive interventions should be strengthened to delay the onset of disabilities of this population. Essentials in delaying the onset of disabilities are controlling disease activity, enhancing functional exercises, strengthening psychological education, and improve quality of life.

## Abbreviations

BMI: Body mass index
BP: Body pain
CRP: C-reactive protein
DAS28: Disease activity score in 28 joints
ESR: Erythrocyte sedimentation rate
GH: General health perception
HADS: Hospital anxiety and depression scale
HAQ-DI: The health assessment questionnaire-disability index
MCS: Mental components summary
MH: Mental health
PCS: Physical components summary
PF: Physical functioning
RA: Rheumatoid arthritis
RE: Role emotional
RF: Rheumatoid factor
RP: Role limitations due to physical problems
SF: Social functioning
SF-36: Short form 36 health survey
SJC: Swollen joint count
TJC: Tender joint count
VAS: Visual analog scale
VT: Vitality
